# Uromodulin and Estrogen

**DOI:** 10.34067/KID.0000000000000259

**Published:** 2023-09-28

**Authors:** Suttira Intapad

**Keywords:** uromodulin, estrogen, kidney, sex difference

Uromodulin (gene name: UMOD) is a glycoprotein uniquely produced in the kidney by cells of the thick ascending limbs of the loop of Henle and early distal convoluted tubules. The uromodulin protein was first discovered in 1873 by Dr. Carlo Rovida.^1^ Dr. Rovida first described a substance formed hyaline cast in the tubular lumen produced by the kidney's tubular cells, which he named it cilindrina. Subsequently, Drs. Igor Tamm and Frank Horsfall Jr. detected this protein in the urine of healthy individuals in 1950, and the name, Tamm–Horsfall, was given.^2^ Then, it was rediscovered and named uromodulin in 1985 by Muchmore and Decker, who isolated this protein with immunomodulatory properties from the urine of pregnant patients.^3^ While its physiologic role remains elusive, there has been steadily increasing information regarding its production, secretion, and role in pathophysiologic states. The association of uromodulin levels, polymorphisms, and mutations in conjunction with acute and chronic forms of kidney disease has been reported. Missense mutations in the UMOD gene cause autosomal dominant tubulointerstitial disease, and single nucleotide polymorphisms of the UMOD gene are also associated with kidney disease.^4^

Uromodulin is secreted in the urine as one of the most abundant, uniquely produced and released, urinary proteins of the kidney. Urinary uromodulin levels represent tubular secretion because it is not filtered (its MW is 85 KDa). Because it is uniquely produced by the kidney, urinary levels are representative of its production. It exhibits bidirectional secretion because it also releases basolaterally into the renal interstitium and circulation.^5^ The synthesis of uromodulin is believed to be dynamic and varies on the basis of physiologic and pathologic stressors. Some physiologic stimuli have been linked with variations in uromodulin production, such as water intake, salt intake, and vasopressin hormone secretion.^4^ A positive correlation has been described between levels of uromodulin and estimations of nephron mass^6^ because this protein is produced exclusively in the kidney tubules. Surprisingly, this association does not hold true within the context of gender comparison, particularly in female patients. Some studies reported higher levels of urinary uromodulin^6,7^ and serum uromodulin^8,9^ in female patients compared with male patients. This is surprising because female patients have smaller kidneys and a lower total number of nephrons than male patients,^10^ suggesting the possibility of other factors related to sex differences in the regulation of uromodulin production. However, the role of sex hormones in uromodulin synthesis, release, and function has not been elucidated.

In this issue, a study by Nanamatsu *et al.*^11^ measured serum uromodulin levels in a cohort of healthy human subjects from samples made available from the Indiana University Biobank. Nanamatsu and colleagues analyzed the serum uromodulin levels according to sex, age, and body mass index. Sex and body mass index were significant predictors of serum uromodulin levels when the model was adjusted for race and age, with female patients showing significantly higher average levels of serum uromodulin than male patients, which further supported the findings of previous studies.^8,9^ Interestingly, when they further tested the role of the female sex hormone estrogen on the uromodulin production at a molecular level using mouse kidney thick ascending limb 2 cells, they found dose-dependent increasing uromodulin mRNA and protein production after incubating the cells with 10 pM–1 nM of 17*β*-estradiol for 24 hours.^11^ These results suggest that female patients have higher levels of uromodulin production because of the estrogen responsiveness of the UMOD gene. However, the question of whether this estradiol stimulation leads to secretion of uromodulin into both serum and urine in female patients is still unclear because this experiment did not provide results of urinary uromodulin between male and female patients in their study groups. It will be interesting to see whether 17*β*-estradiol acts as both a simulator of the synthesis and a regulator of the direction of uromodulin secretion. Another important note is that both male and female patients seem to have declining average values within increasing age ranges (age range: 0–40, 41–60, and 61+ years). However, simple linear regression analysis between serum uromodulin and age in both male and female patients suggests that only female patients exhibit a trend (*P* = 0.053) toward decreasing serum uromodulin with age.^11^ Although this result was not statistically significant, it created the question of whether decreases of serum uromodulin with age in female patients are due to the decline in estrogen production as female patients progress toward menopause. Moreover, the decline in estrogen production and uromodulin with age in female patients may have a direct/indirect effect on the increased incidence of kidney disease in female patients with age that is normally observed in the clinical literature.

In the past decade, researchers and clinicians are increasingly reporting and interpreting urinary and serum uromodulin as biomarkers for disease risk, activity, and prognosis. However, several areas of ambiguity are perpetuated in the literature when the activity of uromodulin is studied without context. These factors are related to the unique physiology of the kidney when studies do not clearly demonstrate how other physiologic stimulators and regulators, such as sex hormones, affect its functions. The influence of sex hormones on biomarkers of kidney function and disease needs to be further investigated because they may significantly affect our interpretation of their levels and significance, for instance, the use of uromodulin as a biomarker of any disease risk among the group of male and female patients and, importantly, the andropause/menopause status of the patient.
